# Clinical Manifestations and Genetic Spectrum of Oculocutaneous Albinism Type 2 in Chinese Patients

**DOI:** 10.3390/genes17050583

**Published:** 2026-05-19

**Authors:** Chonglin Chen, Jun Li, Bingqi Wang, Junyi Liu, Xinping Yu

**Affiliations:** State Key Laboratory of Ophthalmology, Zhongshan Ophthalmic Center, Sun Yat-sen University, Guangdong Provincial Key Laboratory of Ophthalmology and Visual Science, Guangzhou 510060, China; chenchonglin@gzzoc.com (C.C.); lijun@gzzoc.com (J.L.); weidocuddle@163.com (B.W.); ljyyi123@163.com (J.L.)

**Keywords:** oculocutaneous albinism type 2, clinical characteristics, genetic spectrum, foveal hypoplasia

## Abstract

**Objectives**: Oculocutaneous albinism (OCA), a group of inherited disorders characterized by deficient melanin synthesis, leads to hypopigmentation of the skin, hair, and eyes. OCA type 2 (OCA2) is caused by mutations in *OCA2*. This study aimed to characterize the comprehensive clinical and genetic spectrum of *OCA2*, and to identify the key ocular determinants of visual acuity. **Methods**: We enrolled 90 probands clinically diagnosed with albinism. Whole-exome sequencing and comprehensive ophthalmic examinations were performed. **Results**: *OCA2* was confirmed in 29 probands (32.2%). Visual impairment was distributed as mild/no impairment (30.8%), moderate (53.8%), and severe/blindness (15.4%). All patients exhibited nystagmus and photophobia. Ocular phenotype grading showed distinct distributions: iris translucency (*n* = 25) was 68% grade 3, 20% grade 2, 8% grade 1, and 4% grade 4; fundus hypopigmentation (*n* = 26) was 42.3% grade 1, 30.8% grade 2, and 26.9% grade 3; and foveal hypoplasia (*n* = 20) was 70% grade 4, 25% grade 3, and 5% grade 1. We identified 33 *OCA2* variants (26 compound heterozygous and 3 homozygous), with missense variants accounting for 62.1% of alleles. Five variants were identified to be novel. The severity of foveal hypoplasia demonstrated a strong, statistically significant negative correlation with visual acuity (r = −0.71, *p* < 0.001). **Conclusions**: OCA2 accounts for 32.2% of albinism cases, with moderate visual impairment being the most common (53.8%). Graded phenotyping demonstrated moderate-to-severe iris translucency (88%), mild fundus hypopigmentation (42.3%), and severe (grade 4) foveal hypoplasia (70%). The severity of foveal hypoplasia emerged as an important determinant of visual acuity.

## 1. Introduction

Oculocutaneous albinism (OCA), a group of autosomal recessive disorders, results in the deficiency of melanin synthesis and consequent hypopigmentation of the skin, hair, and eyes. Except for the characteristics of light hair and skin, patients with OCA are accompanied by complex and diverse ocular abnormalities, such as poor visual acuity, photophobia, strabismus, nystagmus, iris transillumination, foveal hypoplasia, fundus hypopigmentation, and abnormal decussation of the visual pathways [1].

OCA encompasses a range of subtypes and is now classified into eight subtypes based on the type of genetic mutation associated with pigmentation [2]. To date, seven genes have been found to be associated with the development of OCA: tyrosinase (*TYR*, MIM: 606933), oculocutaneous albinism type 2 (*OCA2*, previously called P gene; MIM:611409), tyrosinase-related protein-1(*TYRP1*; MIM: 115501), solute carrier family 45 member 2 (*SLC45A2*; MIM: 606202), solute carrier family 24 member 5 (*SLC24A5*; MIM: 609802), leucine rich melanocyte differentiation associated (*LRMDA*, also named as *C10orf11*; MIM: 614537), and tyrosinase-related protein-2 (*TYRP2*, also known as *DCT*; MIM: 191275), which was responsible for OCA1 to OCA8, respectively [2,3]. Except for OCA5, as the causative gene has not been identified [4].

Oculocutaneous albinism type 2 is caused by mutations of *OCA2*, which is located on chromosome 15q11.2-q12 and consists of 24 exons. It encodes P-protein, an 838-amino acid polypeptide containing 12 transmembrane-spanning domains, which has been reported to have multiple functions in melanin biosynthesis, including: involvement in molecular transport into melanosomes; regulation of melanosomal pH [5]; modulation of the intracellular transport of tyrosinase [6]; and association with mature melanosomes [7].

OCA2 is a major subtype characterized by broad phenotypic heterogeneity. Although its genetic basis is well established, a detailed, quantitative characterization of the ocular phenotype-particularly the structure- function relationships underlying visual acuity loss- remains incompletely defined. In this study, we performed an integrated clinical and genetic analysis of a Chinese OCA2 cohort. Ophthalmic phenotyping and correlation analyses were employed to identify the principal determinants of visual impairment. Concurrently, expanded genetic screening further elucidated the mutational spectrum of OCA2.

## 2. Materials and Methods

### 2.1. Study Subjects and Clinical Evaluation

This study was approved by the Institutional Review Board of Zhongshan Ophthalmic Center, Sun Yat-sen University and conducted in accordance with the Declaration of Helsinki. Written informed consent was obtained from the adult patients and the parents or guardians of the participants under 18 years old.

Clinical data collected for this study included age at presentation, sex, family history. All individuals diagnosed with albinism underwent a comprehensive ophthalmic examination. This examination consisted of assessment of best-corrected visual acuity (BCVA), measurement of intraocular pressure, evaluation of ocular biometric parameters using the IOLMaster^®^ 700 (Carl Zeiss Meditec AG, Jena, Germany), slit-lamp biomicroscopy, binocular indirect ophthalmoscopy, digital fundus photography (Carl Zeiss Meditec AG), and optical coherence tomography (OCT; Carl Zeiss AG). The clinical diagnostic criteria of OCA were based on previous reports [1].

Visual impairment was categorized according to the World Health Organization (WHO) International Classification of Diseases, 10th Revision (ICD-10) criteria: blindness (BCVA ≤ 0.05 or Snellen equivalent ≤ 20/400), severe visual impairment (BCVA > 0.05 to <0.1; 20/400 to <20/200), moderate visual impairment (BCVA ≥ 0.1 to <0.3; 20/200 to <20/60), and mild or no impairment (BCVA ≥ 0.3; ≥20/60) [8].

Ocular phenotypic grading in patients with albinism was performed using established classification schemes. Iris translucency was graded from 0 to 4 as described by Kruijt et al. [1]: grade 0 (normal), grade 1 (minimal punctuate translucency), grade 2 (diffuse translucency), grade 3 (lens visible around the iris), and grade 4 (complete translucency). Fundus pigmentation was assessed on a scale from 0 to 3: grade 0 (normal pigmentation), grade 1 (choroidal vessels visible in the mid-periphery), grade 2 (vessels visible in the posterior pole but sparing the macula), and grade 3 (vessels visible in the macular region). The severity of foveal hypoplasia (FH) was graded from 1 to 4 based on OCT findings, with higher grades indicating greater anatomical disruption: Grade 1 was characterized by an abnormal but present foveal pit, inner retinal layer extrusion, outer segment (OS) layer lengthening, and outer nuclear layer (ONL) widening. Grade 2 lacked a foveal pit. Grade 3 additionally lacked OS lengthening. Grade 4, the most severe form, also lacked ONL widening [9].

### 2.2. Genetic Analysis

Genomic DNA was extracted from peripheral blood samples obtained from enrolled patients. Whole-exome sequencing (WES) was performed for the probands. The exome was captured using the Twist HumanCore Exome Kit (Twist Bioscience, South San Francisco, CA, USA). and sequenced in paired-end 2 × 150 bp format on the Illumina NovaSeq 6000 platform (Illumina, San Diego, CA, USA). Read alignment to the human reference genome GRCh38 and variant calling were performed using the Sentieon Genomics DNASeq software (Sentieon, Inc., San Jose, CA, USA) pipeline (Sentieon, Inc., San Jose, CA, USA), and variant annotation was performed using ANNOVAR (https://annovar.openbioinformatics.org). Potential pathogenic variants were predicted using SIFT (https://siftdna.org), MutationTaster (https://www.mutationtaster.org), and PolyPhen-2 (https://genetics.bwh.harvard.edu/pph2). Variants with an allele frequency below 0.5% in public population databases were retained for further analysis. Disease and phenotype databases, including Online Mendelian Inheritance in Man, ClinVar, the Human Gene Mutation Database, and the Human Phenotype Ontology, were used for variant interpretation. Variants were classified according to the American College of Medical Genetics and Genomics criteria. Copy number variation (CNV) detection was performed using the R package ExomeDepth v1.1.16, with 30 reference samples of the same sex. CNVs were classified according to the recommendations of the American College of Medical Genetics and Genomics and the Clinical Genome Resource committees. Candidate variants were confirmed by bidirectional Sanger sequencing in the probands and available family members, and co-segregation analysis was performed in available family members for each pedigree.

The identified copy number variation was further validated by quantitative polymerase chain reaction (qPCR). Specific primers were designed using Primer-BLAST (https://www.ncbi.nlm.nih.gov/tools/primer-blast, accessed on 14 May 2026). qPCR reactions were performed in 25-μL volumes containing 12.5 μL of 2× PowerUp SYBR Green Master Mix, 1 μL of forward primer, 1 μL of reverse primer, 5.5 μL of nuclease-free water, and 5 μL of template DNA. Assays were conducted on an ABI Q3 system under the following conditions: 95 °C for 5 min; 40 cycles of 95 °C for 10 s and 60 °C for 40 s with fluorescence collection; followed by melting curve analysis. Primer pairs with 90% to 110% amplification efficiency, a specific melting peak, and no melting peak in the negative control were selected. Test samples and two diploid control samples were analyzed using the comparative Ct method. Each sample–primer combination was analyzed in three independent technical replicates, and the Ct difference among triplicates was controlled within 0.3. Relative copy number was calculated using the ΔΔCt method. Co-segregation analysis within families was performed to validate the pathogenicity of identified variants.

### 2.3. Statistical Analysis

All statistical analyses were performed using SPSS (Statistical Product and Service Solutions, Version 23.0; IBM Corporation, Chicago, IL, USA). Demographic and ocular characteristics were summarized using descriptive statistics, with categorical variables presented as frequencies and percentages. Associations between the severity of visual impairment and each ocular grading parameter (foveal hypoplasia, iris translucency, fundus hypopigmentation) were assessed using Spearman’s rank-order correlation. Results are reported as the correlation coefficient (r) with the corresponding two-sided *p*-value. A *p*-value of less than 0.05 was considered statistically significant.

## 3. Results

### 3.1. Demographic and Clinical Characteristics of Probands with OCA2 Mutations

**Figure 1:**
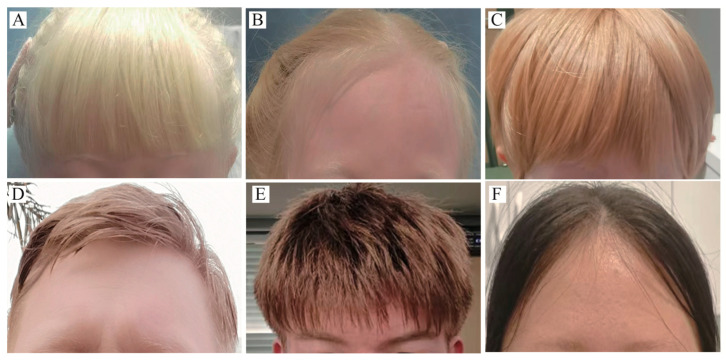
Representative hypopigmentation phenotypes of hair and skin in OCA2 patients. (**A**) A 6-year-old girl exhibiting platinum blond hair. Genetic analysis identified compound heterozygous variants: c.406C > T (p.Arg136Ter), a nonsense variant, and a novel in-frame deletion, c.1560_1562del (p.Leu521del). (**B**) A 6-year-old girl with pale blond hair. She carries compound heterozygous novel variants: a frameshift variant, c.1278delinsTATCAT (p.Met428IlefsTer27), and a missense variant, c.2378G > A (p.Cys793Tyr). (**C**) A 5-year-old girl presenting with blond hair. She is compound heterozygous for two missense variants: c.2363C > T (p.Ser788Leu) and c.2323G > C (p.Gly775Arg). (**D**) A 36-year-old man with light brown hair. Genotyping revealed compound heterozygous missense variants: c.2180T > C (p.Leu727Pro) and c.593C > T (p.Pro198Leu). (**E**) A 17-year-old boy exhibiting dark brown hair. He carries compound heterozygous variants: a missense variant, c.1444A > G (p.Thr482Ala), and the nonsense variant c.406C > T (p.Arg136Ter) also identified in patient A. (**F**) A 36-year-old woman with minimally pigmented, almost black hair. She is compound heterozygous for two missense variants: c.2359G > A (p.Ala787Thr) and c.1079C > T (p.Ser360Phe).

In this study, a total of 90 unrelated probands with a clinical diagnosis of albinism were enrolled. Among these, *OCA2* mutations were genetically confirmed in 29 probands. The mean age was 15.6 ± 12.4 years, with a median age of 10 years (range: 1–41 years). Cutaneous examination revealed that white skin color was the predominant phenotype, present in 26 individuals (89.7%). The remaining cases exhibited reddish-white skin (2/29, 6.9%) or skin with significant residual pigmentation (1/29, 3.4%). Hair color showed broad phenotypic variation, distributed as follows: platinum blond (1/29, 3.4%), pale blond (6/29, 20.7%), blond (7/29, 24.1%), light brown (7/29, 24.1%), dark brown (7/29, 24.1%), and almost black (1/29, 3.4%). (Table 1 and Figure 1)

### 3.2. Ocular Manifestations and Grading in Patients with OCA2 Mutations

**Figure 2:**
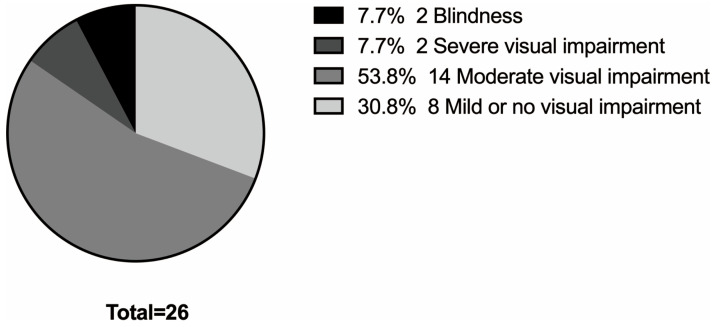
Distribution of visual impairment severity in patients with OCA2. Quantitative visual acuity data were available for 26 out of 29 enrolled patients and are presented: blindness, *n* = 2 (7.7%); severe visual impairment, *n* = 2 (7.7%); moderate visual impairment, *n* = 14 (53.8%); and mild or no visual impairment, *n* = 8 (30.8%).

**Figure 3:**
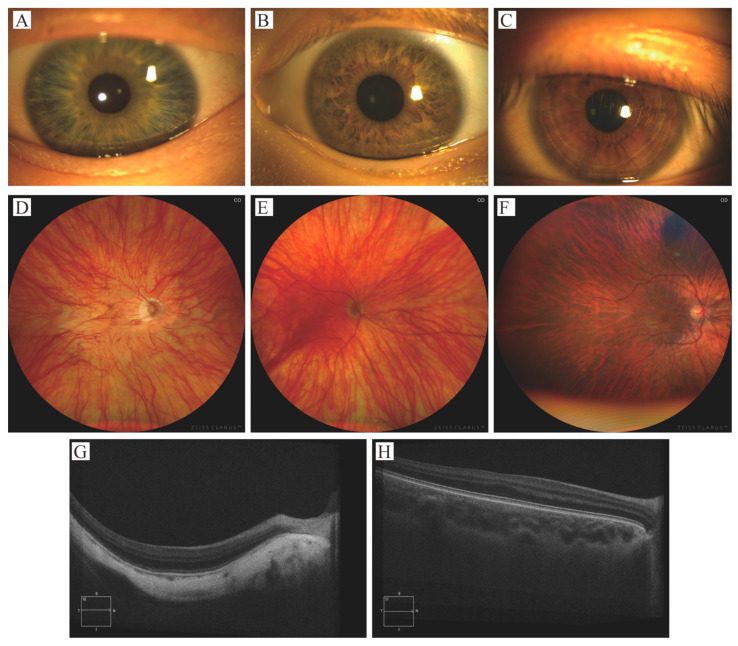
Representative spectrum of ocular phenotypes in patients with OCA2. Images illustrate the variable clinical presentation across three key ocular features. (**A**–**C**) Graded iris translucency, demonstrating progressive visibility of underlying structures: (**A**) grade 3 (lens visible circumferentially), (**B**) grade 2 (diffuse translucency), and (**C**) grade 1 (minimal punctuate translucency). (**D**–**F**) Graded fundus hypopigmentation: (**D**) grade 3 (choroidal vessels visible in the macular region), (**E**) grade 2 (vessels visible in the posterior pole, sparing the macula), and (**F**) grade 1 (vessels visible only in the mid-periphery). (**G**,**H**) Optical coherence tomography scans demonstrating the anatomical spectrum of foveal hypoplasia: (**G**) grade 4 (most severe, with absence of foveal pit, outer segment lengthening, and outer nuclear layer widening) and (**H**) grade 1 (mildest form with a shallow foveal pit and partial preservation of layered architecture).

Among the 29 patients, visual acuity could be quantified in 26 cases. Of these, 8 patients (30.8%) had mild or no visual impairment, 14 (53.8%) had moderate visual impairment, 2 (7.7%) had severe visual impairment, and 2 (7.7%) were classified as blind. (Figure 2) The BCVA ranged from 0.3 to 1.3 logMAR. All 29 patients exhibited photophobia and nystagmus (100%). Strabismus was present in 8 patients (27.6%), with esotropia (5/29, 17.2%) being more common than exotropia (3/29, 10.3%). Iris translucency, assessed in 25 patients, was predominantly grade 3 (17/25, 68%), followed by grade 2 (5/25, 20%), grade 1 (2/25, 8%), and grade 4 (1/25, 4%). Fundus hypopigmentation, evaluated in 26 patients, was most frequently graded as 1 (11/26, 42.3%), followed by grade 2 (8/26, 30.8%) and grade 3 (7/26, 26.9%). Assessment of foveal hypoplasia in 20 patients showed a predominance of severe grade 4 involvement (14/20, 70%), with grade 3 observed in 5 patients (25%) and grade 1 in 1 patient (5%) (Table 2 and Figure 3).

### 3.3. OCA2 Mutations and Genetic Analysis

**Figure 4:**
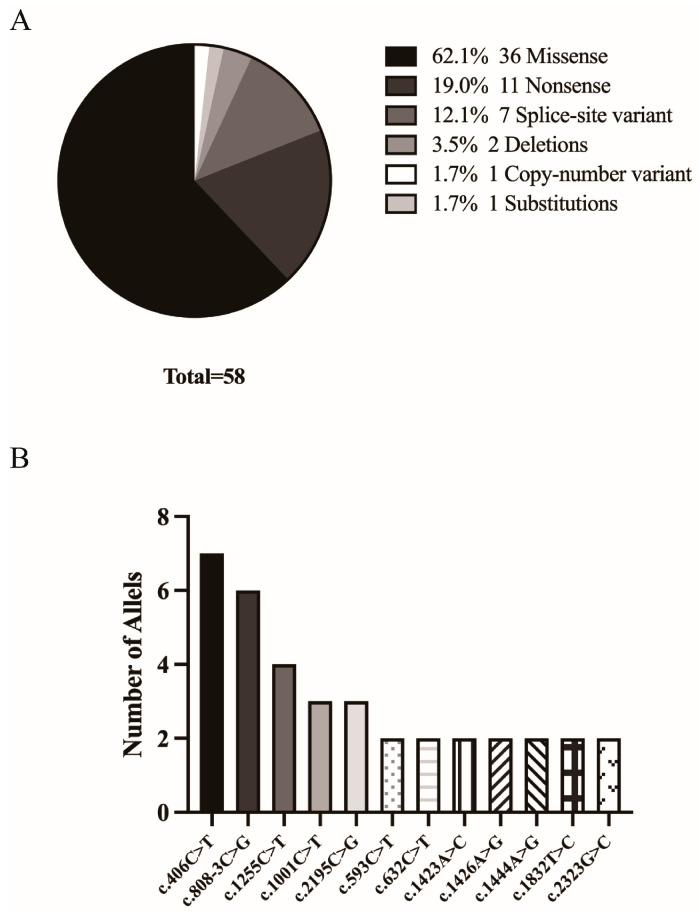
Spectrum of pathogenic *OCA2* variants identified in the cohort. (**A**) Distribution of variant classes among all mutant alleles (*n* = 58). Missense variants predominated (62.1%, 36/58), followed by nonsense (19.0%, 11/58), splice-site (12.1%, 7/58), deletions (3.4%, 2/58), a copy-number variant (1.7%, 1/58), and a substitution (1.7%, 1/58). (**B**) The most prevalent individual alleles, shown as allele count. Only variants detected in ≥2 alleles are displayed. Allele frequencies are: c.406C > T (7), c.808-3C > G (6), c.1255C > T (p.Arg419Trp) (4), c.1001C > T (p.Ala334Val) (3), c.2195C > G (3), c.593C > T (p.Pro198Leu) (2), c.632C > T (p.Pro211Leu) (2), c.1423A > C, (p.Thr475Pro) (2), c.1426A > G (p.Asn476Asp) (2), c.1444A > G (p.Thr482Ala) (2), c.1832T > C (p.Leu611Pro) (2), and c.2323G > C (p.Gly775Arg) (2).

Among the 29 probands, a total of thirty-three distinct variants in the OCA2 gene were identified. Twenty-six patients carried compound heterozygous genotypes, while three were homozygous. At the allele level, the spectrum of variants consisted of missense (36/58, 62.1%), nonsense (11/58, 19.0%), splice-site variant (7/58, 12.1%), deletions (2/58, 3.4%), substitutions (1/58, 1.7%), and one copy-number variant (1/58, 1.7%). (Table 3 and Figure 4).

The nonsense variant c.406C > T was the most frequently identified, found in 5 probands (7 alleles). This was followed by the splice-site variant c.808-3C > G, detected in 6 probands (6 alleles), and the missense variant c.1255C > T (p.Arg419Trp), identified in 4 probands (4 alleles). The nonsense variant c.2195C > G and the missense variant c.1001C > T (p.Ala334Val) were each present in 3 probands (3 alleles). Six missense variants—c.593C > T (p.Pro198Leu), c.632C > T (p.Pro211Leu), c.1426A > G (p.Asn476Asp), c.1444A > G (p.Thr482Ala), c.1832T > C (p.Leu611Pro), and c.2323G > C (p.Gly775Arg)—were each detected in 2 probands (2 alleles). A homozygous pathogenic missense variant c.1423A > C, (p.Thr475Pro) was identified in one proband. All remaining variants were identified in a single proband each. (Figure 4).

In addition, five novel variants were identified. These included two missense variants (c.1210A > G, p.Thr404Ala and c.1288T > C, p.Cys430Arg), an in-frame deletion (c.1560_1562del, p.Leu521del), a frameshift variant (c.1278delinsTATCAT, p.Met428IlefsTer27), and a 0.39 kb copy-number deletion at 15q13.1.

### 3.4. Correlation Analysis Between Visual Acuity and Ocular Phenotypes

**Figure 5:**
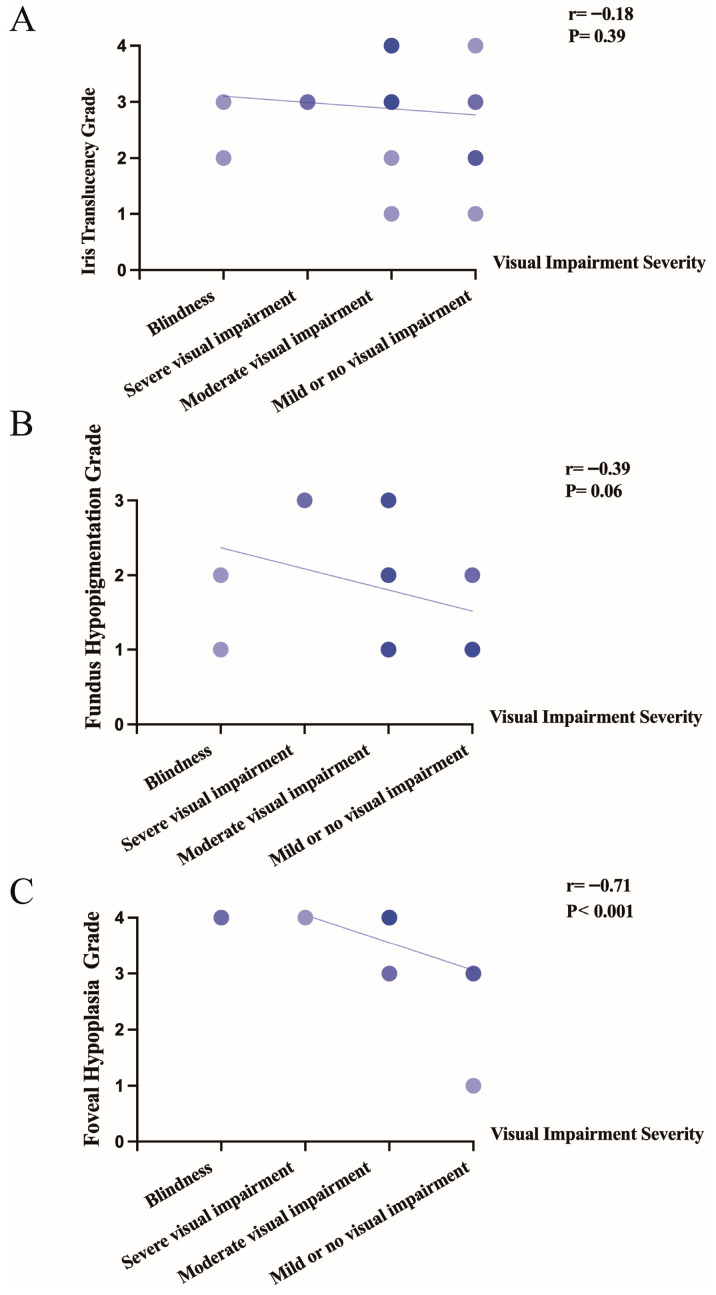
Correlations between visual impairment severity and ocular phenotype grades in OCA2. (**A**) Iris translucency grade versus visual impairment severity: no significant association was observed, showing only a non-significant trend toward a negative correlation (r = −0.18, *p* = 0.39). (**B**) Fundus hypopigmentation grade versus visual impairment severity: no significant association was detected, showing only a non-significant trend toward a negative correlation (r = −0.39, *p* = 0.06). (**C**) Foveal hypoplasia grade versus visual impairment severity: a significant negative association was observed (r = −0.71, *p* < 0.001). The *x*-axis shows categories of visual impairment severity, and the *y*-axis indicates the corresponding clinical grading for each ocular feature. Each dot represents an observation; dot color intensity reflects the number of subjects at the corresponding grade–severity combination, with darker colors indicating higher counts. Solid lines denote fitted trend lines.

To evaluate the association between ocular structural grading and the-WHO-defined severity of visual impairment (blindness, severe, moderate, or mild/no impairment), a stratified correlation analysis was performed. The results showed that neither iris translucency grade (r = −0.18, *p* = 0.39) nor fundus hypopigmentation grade (r = −0.39, *p* = 0.06) was significantly correlated with visual status, both displaying only weak, non-significant negative trends. In contrast, a statistically significant negative correlation was identified between foveal hypoplasia grade and visual impairment severity (r = −0.71, *p* < 0.001), indicating that worse foveal development is directly associated with poorer visual acuity (Figure 5).

## 4. Discussion

The genetic epidemiology of albinism demonstrates substantial geographic and population-specific variation. Globally, OCA2 is a major subtype, estimated to account for approximately 30% of cases [28]. Its prevalence is highest in Africa, affecting an estimated 1 in 10,000 individuals, and as many as 1 in 1000 in certain populations [29]. In specific Southern African groups, a founder 2.7 kb deletion within the OCA2 gene accounts for about 78% of disease-causing mutations [30]. An analysis of a Pakistani cohort with non-syndromic OCA found that variants in TYR (OCA1) and OCA2 together explained the majority of cases, identified in 43/90 (47.8%) and 30/90 (33.3%) of patients, respectively [31]. In contrast, the mutational spectrum in East Asian populations differs significantly. In Japan, OCA4 is the most prevalent subtype (67/290, 23.1%), followed by OCA1 (57/290, 19.7%), and OCA2 (30/290, 10.3%) [32]. Correspondingly, another study from China identified OCA1 as the predominant form (75/114, 65.8%), with OCA2 ranking second (16/114, 14.0%) [15]. In our cohort, the proportion of OCA2 was notably higher, accounting for 32.2% (29/90) of cases. The geographic variation in OCA subtype frequencies highlights the combined impact of population genetics, founder effects, and methodological factors—including case ascertainment criteria and the scope of genetic screening—on the global genetic architecture of albinism.

“Brown” oculocutaneous albinism (OCA) [33], initially reported in Nigerian and Ghanaian families and once considered a separate entity [34], is now understood to belong to the phenotypic spectrum of OCA2-related disease. This spectrum encompasses hypopigmented “classic” forms—typically presenting with blond hair, creamy-tan skin, and blue/hazel irides—as well as more pigmented “brown” phenotypes, also referred to in earlier literature as incomplete or tyrosinase-positive albinism [35]. Consistent with this broad phenotypic continuum, the hair color in our cohort displayed notable heterogeneity, ranging from platinum blond and blond to brown and almost black, further underscoring the diverse expressivity of OCA2.

To date, systematic characterization of ocular features specifically in genetically confirmed OCA2 cohorts remains scarce. A study from Denmark reported the following ocular features in patients with albinism: foveal hypoplasia (95.2%), nystagmus (93.5%), iris translucency (80.2%), and fundus hypopigmentation (75.8%). The study found no significant phenotypic differences across the different genetic subtypes [36]. In a prospective cohort study conducted at Moorfields Eye Hospital involving 44 individuals referred with “suspected albinism,” genetic testing confirmed OCA2 variants in four families. All affected individuals exhibited nystagmus, with a mean best-corrected visual acuity (BCVA) of approximately 0.5 logMAR (range 0.2–1.2) [37]. In our cohort, the BCVA ranged from 0.3 to 1.3 logMAR, with moderate visual impairment being the most common (53.8%). Photophobia and nystagmus were present in all patients. Strabismus was observed in 27.6% of cases, with esotropia constituting the predominant type. Quantitative grading of ocular structures indicated that iris translucency was most commonly moderate to severe (88% combined grades 2–3), fundus hypopigmentation was typically mild (grade 1, 42.3%), and foveal hypoplasia was characteristically severe, with grade 4 involvement in 70% of assessable patients. This combination of functional and structural findings delineates a distinct and clinically consequential ocular phenotype associated with OCA2 mutations.

Pathogenic OCA2 variants impair melanosome function and melanin synthesis, directly causing iris and fundus hypopigmentation. Additionally, aberrant melanin/melanosome-related signaling from the retinal pigment epithelium may disrupt foveal development, leading to foveal hypoplasia with persistent inner retinal layers and incomplete cone specialization [2,35]. To further evaluate structure-function relationships in OCA2, we investigated the association between graded ocular phenotypes and the severity of visual impairment. Notably, foveal hypoplasia grade demonstrated a statistically significant negative correlation with visual acuity (r = −0.71, *p* < 0.001), suggesting that the degree of foveal maldevelopment is a principal determinant of visual deficit in this population. In contrast, neither iris translucency (r = −0.18, *p* = 0.39) nor fundus hypopigmentation (r = −0.39, *p* = 0.06) exhibited a significant association with visual status, implying that these features, while characteristic of the phenotype, are less directly linked to functional vision loss. These findings underscore the importance role of foveal structure in visual prognosis in OCA2 and highlight the importance of detailed macular assessment in clinical management. Foveal hypoplasia appears to be an important intrinsic determinant of BCVA in this cohort, although other clinical factors (e.g., age, refractive error, nystagmus severity, strabismus/amblyopia, and fixation instability) may also contribute.

The pathogenic variant spectrum of OCA2 is notably broad and heterogeneous, encompassing missense, loss-of-function (nonsense, frameshift, splice-site), and copy-number variants [26]. No single mutational hotspot is universal; instead, the spectrum in different populations is shaped by distinct founder effects. This is exemplified by the high frequency of the exon 7 2.7 kb deletion in sub-Saharan Africa [38] and the recurrent c.1327G > A (p.Val443Ile) missense variant in Northern European populations [39]. In our cohort, the allelic distribution was consistent with this diversity. Missense variants predominated (36/58 alleles, 62.1%), followed by nonsense (19.0%), splice-site (12.1%), deletion (3.4%), substitution (1.7%), and copy-number variants (1.7%). The most frequent single allele was the nonsense variant c.406C > T, detected in 7 alleles from 5 probands. Other prevalent alleles included the splice-site variant c.808-3C > G (6 alleles) and the missense variant c.1255C > T (p.Arg419Trp; 4 alleles). Furthermore, we identified five novel variants, underscoring the ongoing expansion of the OCA2 mutational landscape.

This study has several limitations. First, owing to the rarity of albinism, our cohort had a relatively limited sample size. Larger multicenter or population-based studies are therefore needed to validate the genotype distribution and phenotype frequencies observed in this cohort. Second, as a tertiary referral center for complex ocular conditions, our patient population may be subject to selection bias. Third, although OCT parameters were measured repeatedly, the quality and reliability of these measurements can be influenced by fixation instability, which may introduce variability despite our standardized protocol.

## 5. Conclusions

In conclusion, this study provides a comprehensive characterization of the clinical and genetic spectrum of OCA2-related albinism in a Chinese cohort. Clinically, the cohort demonstrated the variable phenotypic spectrum of OCA2, which encompassed a broad range of hair coloration and was further defined by quantitative ocular grading. This assessment revealed a pattern of moderate-to-severe iris translucency (88% combined grades 2–3), predominantly mild fundus hypopigmentation (grade 1, 42.3%), and severe foveal hypoplasia (grade 4 in 70% of cases). Most critically, our correlation analysis established foveal hypoplasia severity as the key correlate of visual impairment, underscoring the central role of macular development in functional vision for OCA2 patients. Genetically, this study delineates a broad mutational spectrum for OCA2, dominated by missense variants (62.1% of alleles), followed by nonsense (19.0%) and splice-site (12.1%) alterations. Compound heterozygosity was the most common inheritance pattern. The most frequent single allele was the nonsense variant c.406C > T, with other recurrent alleles including c.808-3C > G and c.1255C > T (p.Arg419Trp). Furthermore, we identified five novel variants, thereby expanding the known allelic landscape of OCA2 and underscoring the value of comprehensive genetic screening.

## Figures and Tables

**Table 1:** Demographic and clinical characteristics of patients with *OCA2* mutations (N = 29).

Clinical Data	No. of Patients (%)
Gender, *n* (%)	
Male	15 (51.7%)
Female	14 (48.3%)
Age, years	
Mean ± SD	15.6 ± 12.4
Median (Range)	10 (1–41)
Skin, *n* (%)	
White Color	26 (89.7%)
Reddish white	2 (6.9%)
Significant residual pigmentation	1 (3.4%)
Hair color, *n* (%)	
Platinum Blond	1 (3.4%)
Pale blond	6 (20.7%)
Blond	7 (24.1%)
Light Brown	7 (24.1%)
Dark Brown	7 (24.1%)
Almost Black	1 (3.4%)

**Table 2:** Ocular characteristics and grading of patients with *OCA2* mutation.

Parameters	No. of Patients (%)
Photophobia (N = 29)	29 (100%)
Nystagmus (N = 29)	29 (100%)
Strabismus (N = 29)	8 (27.6%)
Esotropia	5 (17.2%)
Exotropia	3 (10.3%)
Iris translucency (N = 25)	
Grade 1	2 (8%)
Grade 2	5 (20%)
Grade 3	17 (68%)
Grade 4	1 (4%)
Fundus hypopigmentation (N = 26)	
Grade 1	11 (42.3%)
Grade 2	8 (30.8%)
Grade 3	7 (26.9%)
Foveal hypoplasia (N = 20)	
Grade 1	1 (5%)
Grade 2	0 (0%)
Grade 3	5 (25%)
Grade 4	14 (70%)

**Table 3:** Pathogenic variants detected in *OCA2*.

ID	Nucleotide Change	Protein Change	Coding Impact	Location (hg38)	Allele Frequency	ACMG	Mutation Taster	PROVEAN	PolyPhen-2	Reference
					(East Asian)					
A003	c.2339-2A > C	p.?	Splicing	chr15:27845054	-	Pathogenic	Deleterious	-	-	[10]
	c.808-3C > G	p.?	Splicing	chr15:28294474	0.0000544	Pathogenic	-	-	-	[11]
A004	c.406C > T	p.Arg136Ter	Nonsense	chr15:28027980	0.000163	Pathogenic	-	-	-	[12]
A009	c.1423A > C	p.Thr475Pro	Missense	chr15:27983425	-	Likely pathogenic	Deleterious	Deleterious	probably damaging	[13]
A013	c.1255C > T	p.Arg419Trp	Missense	chr15:27985173	0.000164	Pathogenic	Benign	Deleterious	probably damaging	[14]
	c.2165del	p.Ile722LysfsTer17	Frameshift	chr15:27871233	-	Pathogenic	-	-	-	[15]
A014	c.1426A > G	p.Asn476Asp	Missense	chr15:27983422	-	Likely pathogenic	Deleterious	Deleterious	probably damaging	[16]
	c.1255C > T	p.Arg419Trp	Missense	chr15:27985173	0.000164	Pathogenic	Benign	Deleterious	probably damaging	[14]
A016	c.646T > C	p.Ser216Pro	Missense	chr15:28022501	-	Likely pathogenic	Deleterious	Deleterious	probably damaging	[17]
	c.632C > T	p.Pro211Leu	Missense	chr15:28022515	0.000163	Pathogenic	Deleterious	Deleterious	probably damaging	[13]
A017	c.1255C > T	p.Arg419Trp	Missense	chr15:27985173	0.000164	Pathogenic	Benign	Deleterious	probably damaging	[14]
	15q13.1 deletion 0.39kb	-	Copy-number variant	-	-	-	-	-		Novel
A022	c.2180T > C	p.Leu727Pro	Missense	chr15:27871218	-	Likely pathogenic	Deleterious	Deleterious	probably damaging	[18]
	c.593C > T	p.Pro198Leu	Missense	chr15:28022554	0.000163	Likely pathogenic	Deleterious	Deleterious	probably damaging	[10]
A030	c.2491G > C	p.Ala831Pro	Missense	chr15:27755414	0.0000544	Likely pathogenic	Benign	Deleterious	possibly damaging	[19]
	c.1444A > G	p.Thr482Ala	Missense	chr15:27983404	0	VUS	Deleterious	Deleterious	probably damaging	[20]
A040	c.1832T > C	p.Leu611Pro	Missense	chr15:27955168	0.000218	Pathogenic	Deleterious	Deleterious	probably damaging	[13]
	c.808-3C > G	p.?	Splicing	chr15:28294474	0.0000544	Pathogenic	-	-	-	[11]
A042	c.2195C > G	p.Ser732Ter	Nonsense	chr15:27871203	-	Pathogenic	-	-	-	[15]
	c.1327G > A	p.Val443Ile	Missense	chr15:27985101	0.000381	Likely pathogenic	Deleterious	Neutral	probably damaging	[21]
A043	c.2195C > G	p.Ser732Ter	Nonsense	chr15:27871203	-	Pathogenic	-	-	-	[15]
	c.1832T > C	p.Leu611Pro	Missense	chr15:27955168	0.000218	Pathogenic	Deleterious	Deleterious	probably damaging	[13]
A044	c.808-3C > G	p.?	Splicing	chr15:28294474	0.0000544	Pathogenic	-	-	-	[11]
	c.1964T > A	p.Ile655Asn	Missense	chr15:27926242	0.000163	VUS	Benign	Deleterious	probably damaging	[22]
A50	c.1001C > T	p.Ala334Val	Missense	chr15:28014819	0	Pathogenic	Deleterious	Deleterious	Probably Damaging	[13]
	c.808-3C > G	p.?	Splicing	chr15:28294474	0.0000544	Pathogenic	-	-	-	[11]
A056	c.1001C > T	p.Ala334Val	Missense	chr15:28014819	0	Pathogenic	Deleterious	Deleterious	Probably Damaging	[13]
	c.632C > T	p.Pro211Leu	Missense	chr15:28022515	0.000163	Pathogenic	Deleterious	Deleterious	probably damaging	[13]
A058	c.1363A > G	Arg455Gly	Missense	chr15:27985065	0.00327	Pathogenic	Deleterious	Deleterious	probably damaging	[13]
	c.808-3C > G	p.?	Splicing	chr15:28294474	0.0000544	Pathogenic	-	-	-	[11]
A064	c.1349C > T	p.Thr450Met	Missense	chr15:27985079	0	Pathogenic	Deleterious	Deleterious	probably damaging	[13]
	c.2323G > C	p.Gly775Arg	Missense	chr15:27851397	-	Pathogenic	Benign	Deleterious	probably damaging	[20]
A065	c.2359G > A	p.Ala787Thr	Missense	chr15:27845032	0	Pathogenic	Deleterious	Deleterious	probably damaging	[13]
	c.1079C > T	p.Ser360Phe	Missense	chr15:27990613	-	Pathogenic	Deleterious	Deleterious	probably damaging	[23]
A068	c.1444A > G	p.Thr482Ala	Missense	chr15:27983404	0	VUS	Deleterious	Deleterious	probably damaging	[20]
	c.406C > T	p.Arg136Ter	Nonsense	chr15:28027980	0.000163	Pathogenic	-	-	-	[12]
A070	c.808-3C > G	p.?	Splicing	chr15:28294474	0.0000544	Pathogenic	-	-	-	[11]
	c.593C > T	p.Pro198Leu	Missense	chr15:28022554	0.000163	Likely pathogenic	Deleterious	Deleterious	probably damaging	[10]
A075	c.2363C > T	p.Ser788Leu	Missense	chr15:27845028	0.0000544	Pathogenic	Deleterious	Deleterious	probably damaging	[13]
	c.2323G > C	p.Gly775Arg	Missense	chr15:27851397	-	Pathogenic	Benign	Deleterious	probably damaging	[20]
A076	c.1857C > G	p.Asp619Glu	Missense	chr15:27951878	-	VUS	Benign	Deleterious	probably damaging	[24]
	c.1426A > G	p.Asn476Asp	Missense	chr15:27983422	-	Likely pathogenic	Deleterious	Deleterious	probably damaging	[16]
A079	c.1210A > G	p.Thr404Ala	Missense	chr15:27986616	-	VUS	Deleterious	Deleterious	probably damaging	Novel
	c.1288T > C	p.Cys430Arg	Missense	chr15:27985140	0	Pathogenic	Deleterious	Deleterious	probably damaging	Novel
A080	c.2195C > G	p.Ser732Ter	Nonsense	chr15:27871203	-	Pathogenic	-	-	-	[15]
	c.1255C > T	p.Arg419Trp	Missense	chr15:27985173	0.000164	Likely pathogenic	Benign	Deleterious	probably damaging	[14]
A084	c.406C > T	p.Arg136Ter	Nonsense	chr15:28027980	0.000163	Pathogenic	-	-	-	[12]
	c.1560_1562del	p.Leu521del	In frame	chr15:27966764	-	Likely Pathogenic	-	-	-	Novel
A085	c.1054A > G	p.Arg352Gly	Missense	chr15:27990638	-	VUS	Benign	Deleterious	probably damaging	[25]
	c.1001C > T	p.Ala334Val	Missense	chr15:28014819	0	Pathogenic	Deleterious	Deleterious	Probably Damaging	[13]
A087	c.1278delinsTATCAT	p.Met428IlefsTer27	Frameshift	chr15:27985150	-	Pathogenic	-	-	-	Novel
	c.2378G > A	p.Cys793Tyr	Missense	chr15:27845013	-	Likely pathogenic	Deleterious	Deleterious	probably damaging	[26]
A089	c.833T > G	p.Leu278Ter	Nonsense	chr15:28247440	-	Pathogenic	-	-	-	[27]
	c.406C > T	p.Arg136Ter	Nonsense	chr15:28027980	0.000163	Pathogenic	-	-	-	[12]
A090	c.406C > T	p.Arg136Ter	Nonsense	chr15:28027980	0.000163	Pathogenic	-	-	-	[12]

VUS: variant of uncertain significance.

## Data Availability

The data presented in this study are available on request from the corresponding author due to privacy restrictions (the data are not publicly available).
